# Supplementary material for: In-silico modelling of the impact of hypertension on the mean transvalvular gradients in aortic stenosis
Source: PLoS One. 2023 Jun 8;18(6):e0286644. doi: 10.1371/journal.pone.0286644 (PMC10249852; doi:10.1371/journal.pone.0286644)
Supplement: S1 File — (DOCX) [file pone.0286644.s001.docx]

**Addendum A: Model development**

In the zero-dimensional computer model of the cardiovascular system, the contractility and pressure-volume relationships of the heart chambers are approximated using the time-varying elastance model of Suga et al.(1) The elastance model simulates the myocardial mechanics using a constitutive pressure-volume relationship which, in turn, is a function of the muscle peak systole and diastole elastances, as seen below in equation 1.

$$\begin{aligned} P_{V}=P_{V,0}+\left( E_{V,d}+\frac{E_{V,s}-E_{V,d}}{2} e\left( t \right) \right)\left( V_{V}-V_{V,0} \right) \#\left( 1 \right) \end{aligned}$$

In equation 1, $P_{V,0}$ (mmHg) is the unstressed ventricular pressure, $V_{V,0}$ (ml) is the unstressed ventricular volume, $E_{V,d}$ (mmHg/ml) is the diastole (minimum) elastance, $E_{V,s}$ (mmHg/ml) is the systole (peak) elastance, $P_{V}$ (mmHg) the instantaneous ventricular pressure (same as LVSP or RVSP) and $V_{V}$ (ml) the instantaneous ventricular volume. In the above equation, $e(t)$ (s) is the time-dependant activation function of the heart muscle and is calculated using equation 2 for the ventricles, where $T_{1}$, $T_{2}$ and $T$ (s) are the times at end systole, end of ventricular relaxation and the heart period.(2)

$$\begin{aligned} e\left( t \right)=\left\{ \begin{matrix} \frac{1-\cos\left( \frac{\pi t}{T_{1}} \right)}{2} & 0\leq t<T_{1} \\ \frac{1+\cos\left( \frac{\pi\left( t-T_{1} \right)}{{(T}_{2}-T_{1})} \right)}{2} & T_{1}\leq t<T_{2} \\ 0 & T_{2}\leq t<T \end{matrix} \right.\#\left( 2 \right) \end{aligned}$$

For the left and right atria, the pressure-volume relationships are calculated similarly to the above ventricle equations, but with one difference, being the activation function, $e(t)$, which is calculated using equation 3. In the equation below, $T_{a}$ is the beginning of the P-wave.(3)

$$\begin{aligned} e\left( t \right)=\left\{ \begin{matrix} 0 & 0\leq t<T_{a} \\ 1-\cos\left( 2\pi\frac{t-T_{a}}{T-T_{a}} \right) & T_{a}\leq t<T \end{matrix} \right.\#\left( 3 \right) \end{aligned}$$

To simulate the instantaneous ventricular volume the mass conservation law is applied as shown in equation 4. The inlet and outlet volume flow rates $Q_{in}$, $Q_{out}$ (ml/s) for each heart chamber is taken from the upstream and downstream components. For example, for the left ventricle the inlet volume flow rate is taken from the mitral valve component, $Q_{MI}$ and the downstream flow rate from the aortic sinus, $Q_{AS}$ as seen in figure 1. The instantaneous atria volumes are calculated similarly.

$$\begin{aligned} \frac{dV_{V}}{dt}=Q_{in}-Q_{out} \#\left( 4 \right) \end{aligned}$$

For the current work, the heart valves are modelled using a standard orifice model (2) which works similarly to an ideal diode. The volume flow rate through a heart valve $Q_{valve}$ is calculated using equation 5.

$$Q_{valve}=\left( \frac{2A_{1}^{2}}{K\rho} \right)^{1/2}A_{r}\sqrt{P_{in}-P_{out}}$$

*Equation 5*

In equation 5, $P_{in},P_{out}$ (mmHg) is the valve upstream and downstream compartment static pressures, $A_{r}$ the valve area opening ratio, $A_{1}$ the valve flow area when fully open, $K$ the valve loss coefficient and $\rho$ the blood viscosity. In the orifice-diode model the valve position is a function of the inlet and outlet static pressures, therefore, $A_{r}$ is calculated as:

$$A_{r}=\left\{ \begin{matrix} 1 & P_{in}\geq P_{out} \\ 0 & P_{in}<P_{out} \end{matrix} \right.$$

For example, if the left ventricular pressure is higher than the left atrium pressure the mitral valve will be closed, and vice versa.

To model the flow and corresponding pressure drops in the circulation loops, sets of ODEs are solved for the various sub compartments in the pulmonary and systemic loops. These compartments are modelled using hydraulic equivalent electrical components such as resistances, inductors, and capacitors. Table 1, below, shows the differential equations for the sinuses, arteries, arterioles, capillaries, and veins for both the systemic and pulmonary loops.

Table 1: Sinus, artery, arteriole, capillary, and vein governing equations

| **Compartment** | **Governing differential equations** |
| --- | --- |
| Sinuses (AS, PS) | $\frac{dP_{AS,PS}}{dt}=\frac{Q_{AO,PO}-Q_{AS,PS}}{C_{AS,PS}}$  $\frac{dQ_{AS,PS}}{dt}=\frac{P_{AS,PS}-P_{SAT,PAT}-R_{AS,PS}Q_{AS,PS}}{L_{AS,PS}}$ |
| Arteries (SAT, PAT) including arterioles (SAR, PAR) and capillaries (SCP, PCP) | $\frac{dP_{SAT,PAT}}{dt}=\frac{Q_{AS,PS}-Q_{SAT,PAT}}{C_{SAT,PAT}}$  $\frac{dQ_{SAT,PAT}}{dt}=\frac{P_{SAT,PAT}-P_{SVN,PVN}-Q_{SAT,PAT}\left( R_{SAT,PAT}+R_{SAR,PAR}+R_{SCP,PCP} \right)}{L_{SAT,PAT}}$ |
| Veins (SVN, PVN) | $\frac{dP_{SVN,PVN}}{dt}=\frac{Q_{SAT,PAT}-Q_{SVN,PVN}}{C_{SVN,PVN}}$  $Q_{SVN,PVN}=\frac{P_{SVN,PVN}-P_{LA,RA}}{R_{SVN,PVN}}$ |

(AS – aortic sinus; PS – pulmonary sinus; SAT – systemic artery; PAT – pulmonary artery; SAR – systemic arteriole; PAR – pulmonary arteriole, SCP – systemic capillaries, PCP – pulmonary capillaries; SVN – systemic veins; PVN – pulmonary veins; dP -change in pressure; dt – change in time; Q – flow; C – Capacitance; P – Pressure; L – Inductance; dQ – change in flow rate)

As seen in the above model equations, there are various empirical inputs required such as heart chamber peak systole elastance $E_{V,s}$, aortic sinus impedance $R_{AS}$ and heart beat period $T$. Tables 2 and 3 below summarises the necessary empirical inputs.(2)

Table 2: Cardiovascular model heart chamber and valve inputs

| **Left and right heart chambers** | | | | | |
| --- | --- | --- | --- | --- | --- |
| **Parameter** | **LV** | **RV** | **LA** | **RA** | **Units** |
| $E_{V,s}$ | 2.5 | 1.15 | - | - | mmHg/ml |
| $E_{V,d}$ | 0.1 | 0.1 | - | - | mmHg/ml |
| $P_{(V,a),0}$ | 1 | 1 | 1 | 1 | mmHg |
| $V_{(V,a),0}$ | 5 | 10 | 4 | 4 | ml |
| $E_{a,max}$ | - | - | 0.25 | 0.15 | ml |
| $E_{a,min}$ | - | - | 0.25 | 0.15 | ml |
| $T_{1}$ | 0.3 | 0.3 | - | - | s |
| $T_{2}$ | 0.45 | 0.45 | - | - | s |
| $T_{a}$ | - | - | 0.8 | 0.8 | s |
| $T$ | 1.0 (s) | | | | |
| **Heart valves** | | | | | |
| **Parameter** | **AO** | **MI** | **PO** | **TI** | **Units** |
| $CQ=\left( \frac{2A_{1}^{2}}{K\rho} \right)^{1/2}$ | 350 | 400 | 350 | 400 | ml/(s mmHg)^0.5^ |

${(E}_{v,s}$- systolic ventricle elastance; $E_{v,d}$ – diastolic ventricle elastance; $P_{\left( V,a \right),0}$ – unstressed ventricle and atrial pressure; $V_{\left( V,a \right),0}$ – unstressed ventricle and atrial volume; $E_{a,max}$ – maximal atrial elastance; $E_{a,min}$ – minimal atrial elastance; CQ – valve flow coefficient)

Table 3: Systemic and pulmonary loop model inputs

| **Systemic loop** | | **Pulmonary loop** | |  |
| --- | --- | --- | --- | --- |
| **Parameter** | **Value** | **Parameter** | **Value** | **Units** |
| $L_{AS}$ | 6.2E-5 | $L_{PS}$ | 5.2E-5 | mmHg s^2^/ml |
| $R_{AS}$ | 0.003 | $R_{PS}$ | 0.002 | mmHg s/ml |
| $C_{AS}$ | 0.08 | $C_{PS}$ | 0.18 | ml/mmHg |
| $L_{SAT}$ | 0.0017 | $L_{PAT}$ | 0.0017 | mmHg s^2^/ml |
| $R_{SAT}$ | 0.05 | $R_{PAT}$ | 0.01 | mmHg s/ml |
| $C_{SAT}$ | 1.6 | $C_{PAT}$ | 3.8 | ml/mmHg |
| $R_{SAR}$ | 0.5 | $R_{PAR}$ | 0.05 | mmHg s/ml |
| $R_{SCP}$ | 0.52 | $R_{PCP}$ | 0.25 | mmHg s/ml |
| $R_{SVN}$ | 0.075 | $R_{PVN}$ | 0.006 | mmHg s/ml |
| $C_{SVN}$ | 1.5 | $C_{PVN}$ | 20.5 | ml/mmHg |

(L_AS_ – Inductance at aortic sinus, L_PS_ – Inductance at pulmonary root, R_AS_ – resistance at aortic sinus, C_AS_ – Capacitance at aortic sinus; L_SAT_ – Inductance at systemic artery; RS_AT_ – resistance at systemic artery; C_SAT_ – capacitance at systemic artery; R_SAR_ – resistance at systemic artery; R_SCP_ – resistance at systemic capillaries; R_SVN_ – resistance at systemic vein; C_SVN_ – capacitance at systemic vein)

By solving the above equations using an appropriate ODE solver, the flow rates and pressures of the modelled compartments can be simulated under various conditions. In the present work the above computer model is applied to investigate the effect of arterial vasodilators on LVSP ($P_{LV}$), MG ($\bar{P_{LV}-P_{AS}}$), ventricular ejection volume ($\int_{0}^{T} Q_{AO}(t)dt$) and in turn *Zva* at varying degrees of aortic stenosis.

**References**

1. Suga H, Sagawa K, Shoukas A. Load Independence of the Instantaneous Pressure-Volume Ratio of the Canine Left Ventricle and Effects of Epinephrine and Heart Rate on the Ratio. Circulation Research. 1973;32(3):314–22.
2. Korakianitis T, Shi Y. Numerical simulation of cardiovascular dynamics with healthy and diseased heart valves. Journal of Biomechanics [Internet]. 2006;16;39(11):1964–82. Available from: <https://linkinghub.elsevier.com/retrieve/pii/S0021929005002927>

3. Bozkurt S. Mathematical modeling of cardiac function to evaluate clinical cases in adults and children. PLOS ONE. 2019;31;14(10):1–20.
