# Supplementary material for: In-silico modelling of the impact of hypertension on the mean transvalvular gradients in aortic stenosis
Source: PLoS One. 2023 Jun 8;18(6):e0286644. doi: 10.1371/journal.pone.0286644 (PMC10249852; doi:10.1371/journal.pone.0286644)
Supplement: Attachment — Submitted filename: Response to Reviewers.docx [file pone.0286644.s002.docx]

19 April 2023

Attention: Vincenzo Lionetti,
Academic Editor
PLOS ONE

To the Editor

Thank you for the opportunity to respond to the excellent comments of the reviewer. We are pleased with the insight and attention to detail that went into the review. The comments are well received, and the revisions add value to the overall paper.

Please find attached our response to the reviewer’s comments. We have also submitted a ‘markup retained’ copy of the manuscript as well as a ‘markup removed’ version via the submission portal as requested.

**Response to reviewers comments:**

1a. Is Figure 1 missing?

It seems that our labelling of the figures led to some confusion. In the submitted version of the manuscript, Figure 1 is the central illustration, and can be seen on the last page of the auto-generated PDF submission document.

To clarify this in the revised manuscript, we have opted to label the central illustration as “Central Illustration”, with subsequent figures numbered sequentially in the order they appear in the paper.

1b. Reference to Figure 3 (now figure 2)

Comments are well received, and the suggested changes have been made.

1c. Reference to Figure 4 (now figure 3)

Thank you for the remarks. The changes have been made as suggested. The reviewer refers to the central illustration in row 231 – please see comment at 1a.

1d. Reference to figure 5 (now figure 4)

An updated version of this figure was uploaded. In the original figure, the peripheral compliance was altered to change the mean flow rate range as part of follow up modeling on this topic. This was incorrectly uploaded with the changed flow rate. The new figure is now in harmony with the flow rate ranges seen in figure 3. Since the flow rate ranges are now in harmony, we have opted not to add the case in figure 3 to figure 4 to avoid confusion.

1e: Reference to figure 6b (now 5b)

An updated version of this figure was uploaded. Similar to in the comments in 1d, the uploaded figure was part of a subsequent modeling where peripheral compliance was altered to assess the effect of higher flow rates and was incorrectly loaded with the higher flow rate. The flow rate range is now in harmony with the other figures and the elastances used in figure 3 (up to a maximum of 2.5).

2. Reference to changes in MG and MFR with AVA’s less than 1.5cm^2^

Indeed, an exponential increase can be expected with progressively smaller valve areas. This is because of the quadratic relationship between the aortic valve area and the subsequent flow. The illustrated valve areas were chosen as they represent important clinical parameters defining a normal valve, a moderately, severely, and very severely stenosed valve.

3. Suggestion to softening certain statements:

- Row 267 – “We have demonstrated that the impact of hypertension on MG is mediated through changes in MFR.

The authors are of the understanding that, at least the predominant mechanism for hypertension induced changes in mean gradient is related to the impact of hypertension on the mean flow rate. This point was argued using first principles (Reference to Line 89-95).

Alternative pathomechanistic pathways through which hypertension influences MG is not known to us and not illustrated in the current work. However, it remains unclear whether acute changes in blood pressure affects aortic sinus diameter acutely, and given this uncertainty, the sentence was modified as suggested.

- Row 226 – “It is demonstrated that LV elastance has no direct bearing on the MG”

This sentence, as stated, could lead to misconceptions, and was modified to reflect the fact that LV elastance appears to have no independent impact on MG, other than through impacting the MFR, and hence the MG. In other words, if a compensatory mechanism were to keep the MFR constant, we would not expect a change in elastance to have a bearing on the MG.

- Row 293 - Changes in BP mediates its effect on MG via changes in MFR

See comment at row 267. This sentence was similarly amended.

- Row 306 - Sudden expansion losses are the most important component of $\kappa$ , and is influenced by the ratio of the true hydraulic valve area to the sinus dimension. In the current work we have demonstrated the impact of changes in sinus dimension and valve morphology on the expected MG - MFR relationship (Row 304 – 307)

We are not sure what the reviewer suggested to change in the statement. The statement in the first line was supported by a previous publication (referenced in the manuscript). The subsequent sentence refers to study findings that are supported by the current data.

We hope that this brings clarification to the questions raised and again thank the reviewer for his insights, which have improved the document.

Your sincerely

J. Liebenberg
